# Supplementary figures and images for: Larval surveys reveal breeding site preferences of malaria vector Anopheles spp. in Zanzibar City
Source: PLoS One. 2025 May 16;20(5):e0313248. doi: 10.1371/journal.pone.0313248 (PMC12083835; doi:10.1371/journal.pone.0313248)

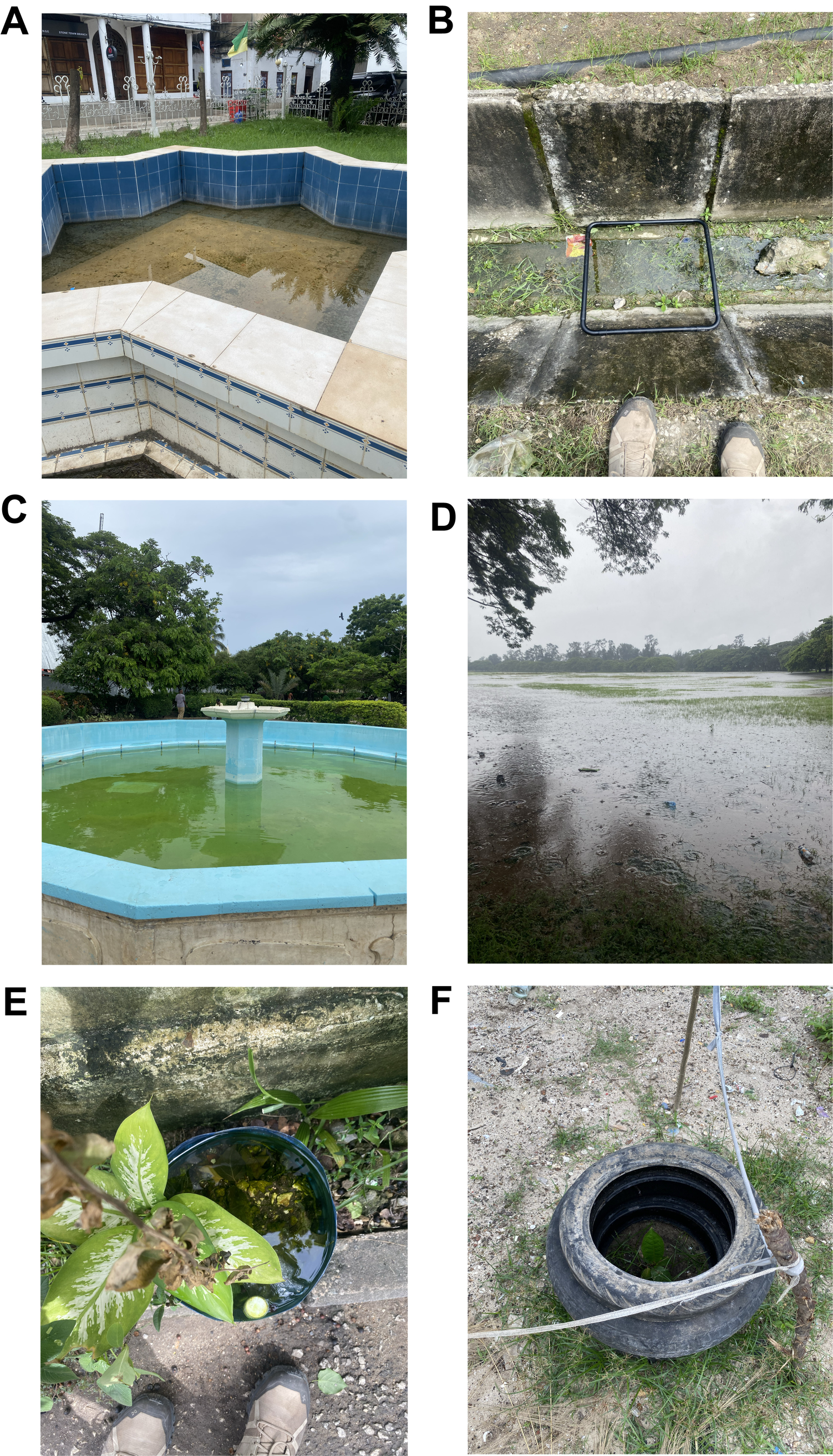

Supplement: S1 Fig — A) Artificial pond. B) Ditch. C) Fountain. D) Wetland. E) Temporary water jug. F) Temporary tire. (TIF) [file pone.0313248.s001.tif]

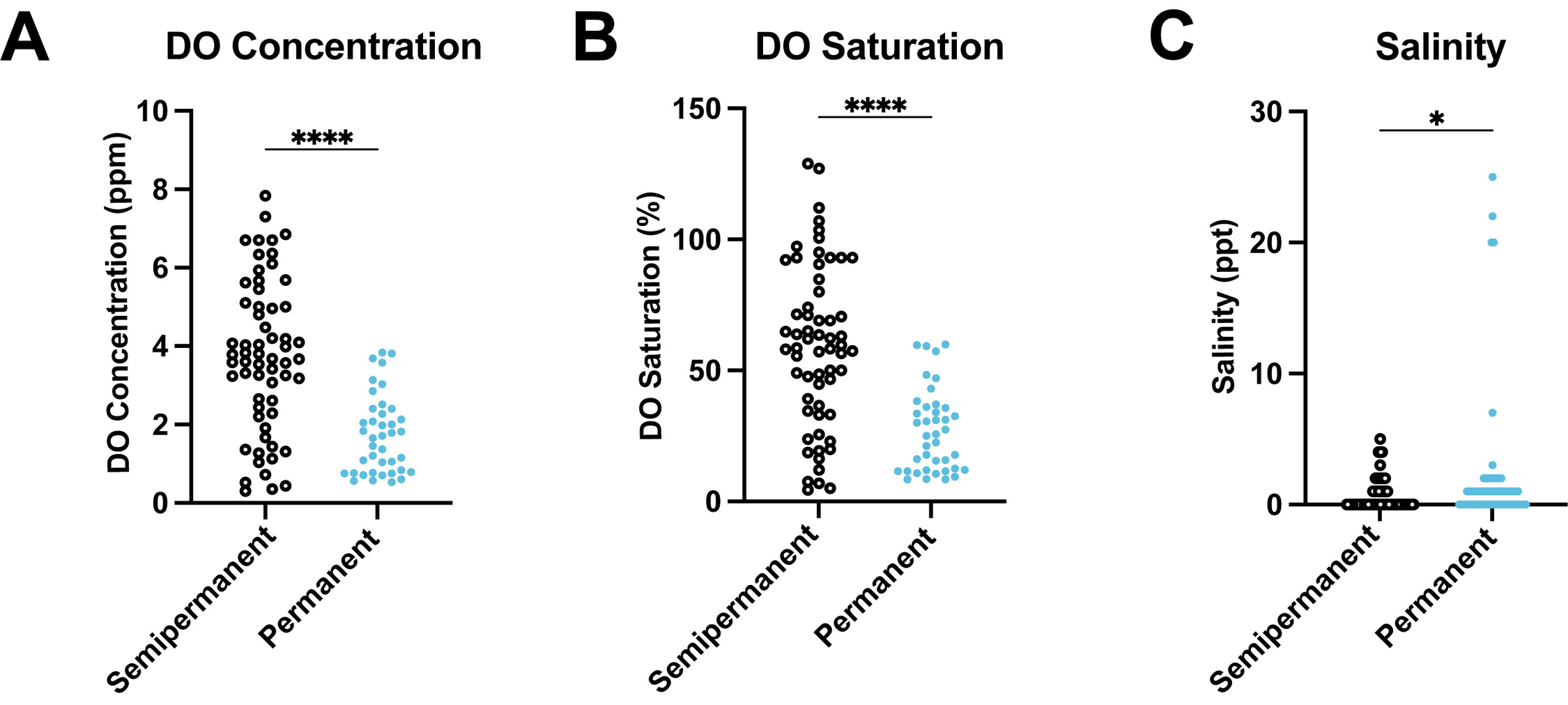

Supplement: S2 Fig — A) Dissolved oxygen concentrations in semi-permanent and permanent subsites. B) Dissolved oxygen saturation percentages in semi-permanent and permanent subsites. C) Salinity levels in semi-permanent and permanent subsites. Statistical significance determined using Student’s unpaired T tests or Mann-Whitney tests for nonparametric data (salinity levels). For all pairwise comparisons: * P < 0.05, **P < 0.01, ***P < 0.001, ****P < 0.0001. (TIF) [file pone.0313248.s002.tif]

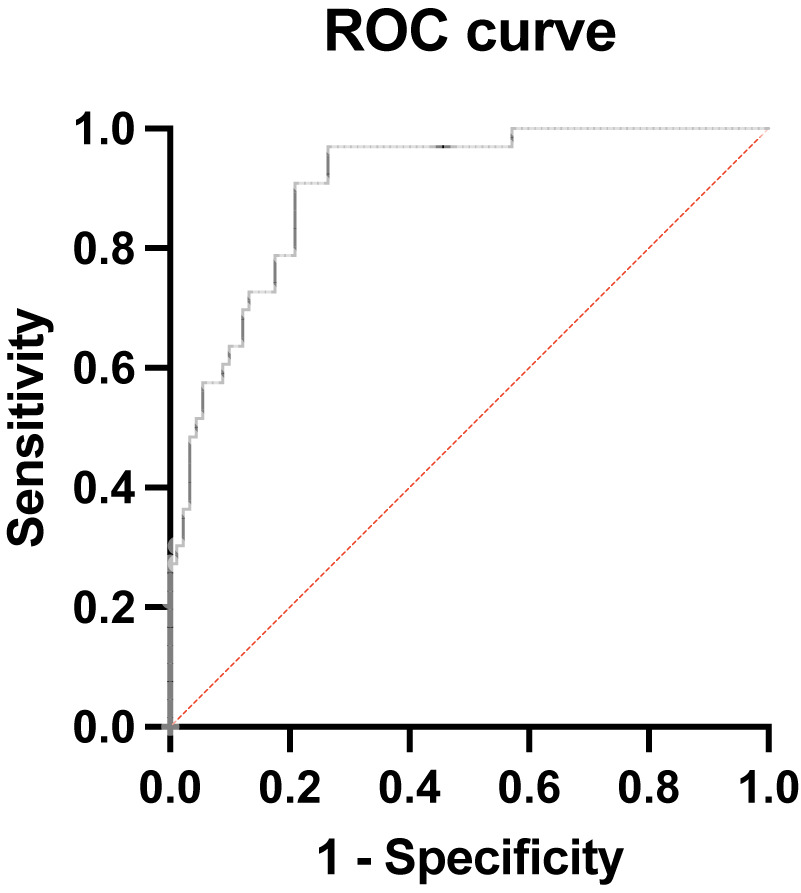

Supplement: S3 Fig — Curve shows a deviation of the current model (black line) from a model with 50/50 odds of predicting Anopheles presence (red line). (TIF) [file pone.0313248.s003.tif]
