# Supplementary material for: Larval surveys reveal breeding site preferences of malaria vector Anopheles spp. in Zanzibar City
Source: PLoS One. 2025 May 16;20(5):e0313248. doi: 10.1371/journal.pone.0313248 (PMC12083835; doi:10.1371/journal.pone.0313248)
Supplement: S1 Table — (PDF) [file pone.0313248.s004.pdf]

S1 Table. Permanent/semi-permanent site-level *Anopheles* larvae abundances.

Site	Site Type	# Anopheles Visit 1	# Anopheles Visit 2
1	Artificial Pond	8	8
2	Artificial Pond	0	0
3	Artificial Pond	4	1
4	Artificial Pond	9	2
5	Ditch	0	1
6	Ditch	0	0
7	Ditch	3	23
8	Ditch	0	0
9	Fountain	0	0
10	Fountain	0	1
11	Fountain	0	38
12	Wetland	0	0
13	Wetland	0	18
14	Wetland	0	2
15	Wetland	0	0
16	Wetland	0	0
