# Supplementary material for: Larval surveys reveal breeding site preferences of malaria vector Anopheles spp. in Zanzibar City
Source: PLoS One. 2025 May 16;20(5):e0313248. doi: 10.1371/journal.pone.0313248 (PMC12083835; doi:10.1371/journal.pone.0313248)
Supplement: S2 Table — All slope estimates are presented in terms of the parameters, not in terms of principal components. Statistical significance for each parameter determined automatically using a T-distribution. Overall significance for the regression determined using ANOVA. For all statistical comparisons: * P < 0.05, **P < 0.01, ***P < 0.001, ****P < 0.0001. (PDF) [file pone.0313248.s005.pdf]

S2 Table. Principal component regression results for physical parameters.

Parameter estimates	Variable	Estimate	P-value	P-value summary
β0	Intercept	8.809	0.3014	ns
β1	% Dips with *Culex*	-0.01914	0.0206	*
β2	% Dips with *Aedes*	-0.02653	0.0223	*
β3	Outdoor Temp (˚C)	-0.1449	0.4581	ns
β4	Water Temp (˚C)	-0.1332	0.4283	ns
β5	% Veg Cover	-0.02336	0.0349	*
β6	Large Dump within 3m	-1.813	0.0399	*
β7	Trash within 3m	-0.5038	0.2153	ns
β8	Depth (cm)	0.02395	0.1812	ns
β9	Predator in Quadrat	0.6232	0.0771	ns
β10	Site Perimeter	0.3423	0.1284	ns
β11	(Semi)permanent	2.394	0.0209	*
β12	Artificial	1.847	0.0311	*
β13	Concrete	2.874	0.0034	**
β14	Plastic/Rubber	-2.425	0.0193	*
β15	Natural	-1.847	0.0311	*
β16	Visit	1.393	0.06	ns
β17	Rain within 48hrs	0.01728	0.3501	ns
Regression ANOVA F Statistic		3.288	0.0308	*

All slope estimates are presented in terms of the parameters, not in terms of principal components. Statistical significance for each parameter determined automatically using a T-distribution. Overall significance for the regression determined using ANOVA. For all statistical comparisons: * *P*<0.05, ***P* <0.01, ****P*<0.001, *****P*<0.0001.
