# Supplementary material for: Larval surveys reveal breeding site preferences of malaria vector Anopheles spp. in Zanzibar City
Source: PLoS One. 2025 May 16;20(5):e0313248. doi: 10.1371/journal.pone.0313248 (PMC12083835; doi:10.1371/journal.pone.0313248)
Supplement: S3 Table — (PDF) [file pone.0313248.s006.pdf]

S3 Table. Predatory taxa found at each site.

		Odonata	Coleoptera	Hemiptera	Plecoptera	Ephemeroptera	Diptera	Fish
1	Artificial Pond	X		X	X			
2	Artificial Pond				X			X
13	Artificial Pond		X	X	X			
15	Artificial Pond	X			X			
8	Ditch			X				
11	Ditch							
14	Ditch	X			X	X	X	
16	Ditch	X			X			
4	Fountain	X		X				
10	Fountain							
12	Fountain							
7-30	Rain Collect.				X			
3	Wetland	X		X				
5	Wetland	X		X		X		
6	Wetland	X	X	X				
17	Wetland	X		X				
18	Wetland	X	X	X	X			X
