# Supplementary material for: Larval surveys reveal breeding site preferences of malaria vector Anopheles spp. in Zanzibar City
Source: PLoS One. 2025 May 16;20(5):e0313248. doi: 10.1371/journal.pone.0313248 (PMC12083835; doi:10.1371/journal.pone.0313248)
Supplement: S4 Table — Statistical significance determined using Chi-square test for independence. (PDF) [file pone.0313248.s007.pdf]

S4 Table. Differences in predator abundance between semi-permanent and permanent subsites.

Site Type	No Predator in Quadrat	Predator in Quadrat	Total
Semipermanent	48 (57.1%)	36 (42.9%)	84 (100%)
Permanent	24 (38.1%)	39 (61.9%)	63 (100%)
Total	72	75	147
X^2	5.227		
P-value	0.0222 (*)		

Statistical significance determined using Chi-square test for independence.
