# Supplementary material for: Larval surveys reveal breeding site preferences of malaria vector Anopheles spp. in Zanzibar City
Source: PLoS One. 2025 May 16;20(5):e0313248. doi: 10.1371/journal.pone.0313248 (PMC12083835; doi:10.1371/journal.pone.0313248)
Supplement: S5 Table — Only significant pairwise comparisons shown. (PDF) [file pone.0313248.s008.pdf]

S5 Table. Two-tailed Mann-Whitney U test results comparing oxygen concentration between subsites with and without *Anopheles* larvae, split by site type.

Mann-Whitney U test	U	Mean Rank Difference	P-value	P-value Summary
Artificial Pond	22.0	-5.556	0.038128	*
Ditch	27.0	-10.95	0.002242	**
Fountain	11.0	7.381	0.008282	**

Only significant pairwise comparisons shown.
