# Supplementary material for: Larval surveys reveal breeding site preferences of malaria vector Anopheles spp. in Zanzibar City
Source: PLoS One. 2025 May 16;20(5):e0313248. doi: 10.1371/journal.pone.0313248 (PMC12083835; doi:10.1371/journal.pone.0313248)
Supplement: S6 Table — Only significant pairwise comparisons shown. (PDF) [file pone.0313248.s009.pdf]

S6 Table. Two-tailed Mann-Whitney U test results comparing oxygen saturation between subsites with and without *Anopheles* larvae, split by site type.

Mann-Whitney U test	U	Mean Rank Difference	P-value	P-value Summary
Ditch	25.0	-11.29	0.001529	**
Fountain	9.00	7.857	0.004438	**

Only significant pairwise comparisons shown.
