# Supplementary material for: Larval surveys reveal breeding site preferences of malaria vector Anopheles spp. in Zanzibar City
Source: PLoS One. 2025 May 16;20(5):e0313248. doi: 10.1371/journal.pone.0313248 (PMC12083835; doi:10.1371/journal.pone.0313248)
Supplement: S7 Table — Only significant pairwise comparisons shown. (PDF) [file pone.0313248.s010.pdf]

S7 Table. Kruskal-Wallace test results comparing dissolved oxygen concentration at subsites with Anopheles between different site types.

Kruskal-Wallace Test	H = 16.18	Groups = 4	*P* = 0.001	
**Dunn's Test for Multiple Comparisons**	**Z**	**Mean Rank Diff.**	**P-value**	**P-value Summary**
Artificial Pond vs. Fountain	2.326	10.39	0.0200	*
Artificial Pond vs. Wetland	3.860	18.33	0.0001	***
Ditch vs. Wetland	2.087	10.48	0.0369	*

Only significant pairwise comparisons shown.
