# Supplementary material for: Larval surveys reveal breeding site preferences of malaria vector Anopheles spp. in Zanzibar City
Source: PLoS One. 2025 May 16;20(5):e0313248. doi: 10.1371/journal.pone.0313248 (PMC12083835; doi:10.1371/journal.pone.0313248)
Supplement: Attachment — Submitted filename: Response-To-Reviewers-02242025.docx [file pone.0313248.s015.docx]

**“Larval surveys reveal breeding site preferences of malaria vector *Anopheles* spp. in Zanzibar City”: Rebuttal Letter**

*Thank you for the opportunity to respond to reviewers’ comments in a revised submission of our manuscript. We thank the reviewers for constructive comments which we have now addressed. The feedback incorporated from reviewers has resulted in an improved version of the manuscript. Line numbers refer to those in the revised manuscript with track-changes.*

**Journal/Editor Requirements:**

1. *“Please ensure that your manuscript meets PLOS ONE's style requirements”*

**Response:** We have reformatted the manuscript such that it is in accordance with the style guidelines.

2. *“Please include a complete copy of PLOS’ questionnaire on inclusivity in global research in your revised manuscript.”*

**Response:** We have completed the questionnaire and included it in our resubmission as S1 File.

3. *“Please remove any funding-related text from the manuscript”*

**Response:** We have removed the funding section from the manuscript.

4. *“We require you to either (1) present written permission from the copyright holder to publish these figures specifically under the CC BY 4.0 license, or (2) remove the figures from your submission.”*

**Response:** We have replaced those figures with maps made based on shapefiles from the Tanzania National Bureau of Statistics (1, 2). The shapefiles provided by the NBS are available for use under this license agreement:

<https://www.nbs.go.tz/nbs/takwimu/references/Licence-Agreement-NBS.pdf>

5. *“We notice that your supplementary figures are uploaded with the file type 'Figure'. Please amend the file type to 'Supporting Information'.”*

**Response:** We have changed the file names.

6. *“We notice that your supplementary tables are included in the manuscript file. Please remove them and upload them with the file type 'Supporting Information'.”*

**Response:** We have removed supporting tables from the manuscript file and uploaded each as supporting information.

**Reviewer 1:**

1. *“Article needs to address grammatical and typographical errors thoroughly. At many places the meaning of the sentences is not clear. Such sentences can be reframed or break into two separate sentences for better readability.”*

**Response:** We have shortened sentences throughout the manuscript to promote better readability.

2. *“The conclusion could be strengthened by briefly outlining the practical implications of the findings for public health policy and future research.”*

**Response:** We thank the reviewer for this suggestion. We have updated the conclusion to address the value of future studies aiming to characterize the seasonal variability of *Anopheles* breeding preferences and determine the productivity of each breeding site. Additionally, we restructured the conclusion to end with a brief list of potential policies (supported by our data) that might help reduce *Anopheles* breeding in Zanzibar City.

3. *“In the discussion, 3-4 sentences on biocontrol agents for mosquito larvae can be included while addressing the study's limitations. Few relevant references can be cited in the study:*
   a. *How effective are Mesocyclops aspericornis (Copepoda: Cyclopoida) in controlling mosquito immatures in the environment with an application of phytochemicals? R Dhanker, R Kumar, JS Hwang, Hydrobiologia 716, 147-162*
   b. *Larvicidal efficiency of aquatic predators: a perspective for mosquito biocontrol - JSH Ram Kumar, Zoological Studies 45 (4), 447”*

**Response:** We thank the reviewer for this suggestion and for these sources. We have incorporated these sources and added a more in-depth discussion of how predators could be used alongside other strategies to control mosquito larvae numbers. As we did not assess copepod abundance in our study, we stress that copepods should be identified alongside other predators during future larval surveys. To better inform future larval surveys or larvicidal strategies in Zanzibar City, we have provided a more detailed depiction of the types of mosquito larvae predators observed in our study (shown below and added to the manuscript as S3 Table).

**S3 Table. Predatory taxa found at each site**

|  |  | Odonata | Coleoptera | Hemiptera | Plecoptera | Ephemeroptera | Diptera | Fish |
| --- | --- | --- | --- | --- | --- | --- | --- | --- |
| 1 | Artificial  Pond | X |  | X | X |  |  |  |
| 2 | Artificial  Pond |  |  |  | X |  |  | X |
| 13 | Artificial  Pond |  | X | X | X |  |  |  |
| 15 | Artificial   Pond | X |  |  | X |  |  |  |
| 8 | Ditch |  |  | X |  |  |  |  |
| 11 | Ditch |  |  |  |  |  |  |  |
| 14 | Ditch | X |  |  | X | X | X |  |
| 16 | Ditch | X |  |  | X |  |  |  |
| 4 | Fountain | X |  | X |  |  |  |  |
| 10 | Fountain |  |  |  |  |  |  |  |
| 12 | Fountain |  |  |  |  |  |  |  |
| 7-30 | Rain  Collect. |  |  |  | X |  |  |  |
| 3 | Wetland | X |  | X |  |  |  |  |
| 5 | Wetland | X |  | X |  | X |  |  |
| 6 | Wetland | X | X | X |  |  |  |  |
| 17 | Wetland | X |  | X |  |  |  |  |
| 18 | Wetland | X | X | X | X |  |  | X |

**Reviewer 2:**

1. *“Many sentences are verbose need restructuring”*

**Response:** We have shortened sentences throughout the article (please see tracked changes document).

2. *“Line 30-44: unnecessary background information not required in abstract. These dilute the essence of present results.”*

**Response:** We thank the reviewer for this suggestion. We have shortened the abstract by removing the first three sentences that introduced the reader to malaria, as this information is obtained in the introduction. The updated abstract begins by telling the reader why larval surveys are needed given the rising trend in malaria cases in Zanzibar City.

3. *“Line 33: vector is not used as verb better to say transmitted”*

**Response:** This sentence was removed from the abstract.

4. *“Line 36-39: Restructure”*

**Response:** We have restructured these lines of the abstract to minimize complex sentence structure.

5. *“Line 57-59 Too broad and too general statement not required in such data riven paper.”*

**Response:** We have restructured this sentence to only include background information on how malaria is transmitted. We want to ensure that this information is presented to readers, as it is crucial to understand the argument of our paper.

6. *“Line 63: Understanding the ecology -" shift after line 74.”*

**Response:** We thank the reviewer for this suggestion. The sentence on line 63 is now at line 68, after we introduce the need for alternative antimalarial strategies.

7. *“Line 79-80: replace which type of breeding sites with attributed of breeding sites and hydro-period”*

**Response:** We have restructured this line.

8. *“Line 83 to 118 These explanation should be provided in discussion and some parts in Introduction before line 79 -i.e. before stating the goal of this paper as Therefore, the aim of this study was to determine which type of breeding sites are used by Anopheles mosquitoes during the rainy season in Zanzibar City and what characterizes them by identifying the physicochemical parameters.”*

**Response:** The introduction has been restructured to address this comment. We now discuss the aims of our study in the last paragraph of the introduction.

9. *“Discussion need reorientation focusing major results, inferences and concurring predators i.e. larvicidal organisms in these habitats, see following papers for larvicidal organisms*
   a. *DOI: 10.1186/s40555-015-0132-9*
   b. *How effective are Mesocyclops aspericornis (Copepoda: Cyclopoida) in controlling mosquito immatures in the environment with an application of phytochemicals? October 2013 , Hydrobiologia*
   c. *Potential of three aquatic predators to control mosquitoes in the presence of alternative prey: A comparative experimental assessment, January 2008; Marine and Freshwater Research 59(9). DOI: 10.1071/MF07143*
   d. *Larvicidal efficiency of aquatic predators: A perspective for mosquito biocontrol. 2006 Zoological Studies*
   e. *Predation on Mosquito Larvae by Mesocyclops thermocyclopoides (Copepoda: Cyclopoida) in the Presence of Alternate Prey 2003 Internationale Revue der gesamten Hydrobiologie und Hydrographie 88(6):570 – 581*
   f. *DOI: 10.1002/iroh.200310631”*

**Response:** We thank the reviewer for this comment and for providing these sources. We have incorporated these sources into the end of our discussion. Additionally, as mentioned in our response to Comment 3 from Reviewer 1, we have provided more detailed data on the types of predators identified at each site. This table has been added to the manuscript as S3 Table, and the results section has been updated accordingly.

10. *“Further discussion should flow around hydro-period, and larval-pupal period in water and breeding by adult Anopheles.”*

**Response:** We thank the reviewer for this suggestion. In addition to our preexisting paragraph on how rain could be either harmful or beneficial to Anopheles breeding (lines 522-539), we have added a discussion on the role that seasonal weather patterns (i.e. hydro-period) might have in Anopheles breeding habits (lines 514-521). This addition to our discussion also addresses your suggestion to further comment on adult *Anopheles* breeding patterns. We cite a recent study showing that the degree of urbanization might affect the extent that hydro-period influences Anopheles breeding patterns (3). The findings presented in that study may be applicable to Zanzibar City, given its rapidly increasing degree of urbanization. As we did not analyze the abundance of *Anopheles* pupae in our study, we have included this shortcoming in our discussion of the study’s limitations (line 757-760). Additionally, we added a note on the importance of conducting future studies on *Anopheles* pupae and adult abundance in order to determine which breeding sites are most effective. We also did not analyze the habits of adult *Anopheles* mosquitoes in our study, and we addressed this limitation in our discussion (lines 588-592) and conclusion (lines 626-628).

Also, as an additional avenue of further discussing breeding by adult *Anopheles*, we have added the possibility that *Anopheles* adults may be able to avoid breeding in sites with high predator abundance through chemical and visual cues (lines 556-559) (4). Therefore, it is crucial for future studies to analyze the dynamics and behavior of adult *Anopheles* mosquitoes in Zanzibar City to design the most effective larvicidal strategy.

11. “*Table 1 is very lengthy only significant values may be shown.”*

**Response:** We have removed non-significant parameters from the table for conciseness.

12. *“Line 610-612: environmentally friendly is very broad and vague statement so explicitly mention what control strategy is recommended by this study , operational control programm, ecology of breeding habitat and recurrence of malaria and”*

**Response:** We have removed the term “environmentally friendly.” Instead, we recommend “introducing phytochemical larvicides into artificial ponds, ensuring that fountains are functional, and preventing the flooding of wetlands (lines 629-632).

**References**

1.	Tanzania Districts Shapefiles 2019. (2012).

2.	2022 Population and Housing Census - Tanzania Wards. (2024).

3.	C. Whittaker, *et al.*, Seasonal dynamics of Anopheles stephensi and its implications for mosquito detection and emergent malaria control in the Horn of Africa. *Proceedings of the National Academy of Sciences* 120, e2216142120 (2023).

4.	R. Kumar, J.-S. Hwang, Larvicidal efficiency of aquatic predators: A perspective for mosquito biocontrol. *Zoological Studies* 45, 447–466 (2006).
