# Supplementary material for: Larval surveys reveal breeding site preferences of malaria vector Anopheles spp. in Zanzibar City
Source: PLoS One. 2025 May 16;20(5):e0313248. doi: 10.1371/journal.pone.0313248 (PMC12083835; doi:10.1371/journal.pone.0313248)
Supplement: Attachment — Submitted filename: Response-to-reviewers-2.docx [file pone.0313248.s016.docx]

**Larval surveys reveal breeding site preferences of malaria vector *Anopheles* spp. in Zanzibar City**

**Response to Reviewers: April 9, 2025**

We would like to thank the editor and reviewers for their thoughtful and thorough feedback. We believe that the additional input from the reviewers has further strengthened our manuscript.

**Editor:**

*Please review your reference list to ensure that it is complete and correct. If you have cited papers that have been retracted, please include the rationale for doing so in the manuscript text, or remove these references and replace them with relevant current references. Any changes to the reference list should be mentioned in the rebuttal letter that accompanies your revised manuscript. If you need to cite a retracted article, indicate the article’s retracted status in the References list and also include a citation and full reference for the retraction notice.*

**Response:** We have screened our entire reference list against a list of retracted articles, and we did not find that any of the cited articles were retracted. We have corrected a few minor formatting errors within the reference lists, including html code remaining in a title, extra periods, and incorrectly formatted author names. Our reference list is now fully corrected.

**Reviewer #1:**

*The authors have thoroughly addressed all the queries. The manuscript can be accepted after removing the citations from the conclusion, as citations are generally not included in this section.*

**Response:** We thank the reviewer for this comment. We have removed citations from the conclusion. No new material was referenced in the conclusion.

**Reviewer #2:**

*Line 220 221 Data analysis and statistics: better change to statistical analyses*

**Response:** We thank the reviewer for this comment. We have updated the title of this subsection.

*Line 222: delete was as “All statistical analyses were done using GraphPad Prism 10 (10.1.1).*

**Response:** We thank the reviewer for catching this error in our phrasing. We have updated the sentence.

*Table -1 Delete the last column i.e. P-value summary; p values are given very clearly no need to add additional column for summary*

**Response:** We have removed this column from Table 1 in the manuscript.

*Line 522: In correct: Because this sampling period took place during the April rainy season. Correct: Because this sampling event took place during the April i.e. in rainy season*

**Response:** We thank the reviewer for this suggestion. We have updated this sentence.

*Line 528-537 : Rephrase sentence and split in two sentences: Of all the sites sampled in this study, those most subjected to drainage coming into the site (and potentially bringing Anopheles larvae from other breeding sites) were those classified as drains and the wetland located at the eastern extreme of the study area.*

**Response:** We thank the reviewer for this suggestion. We have updated these sentences.
